# Endometrial Mesenchymal Stem Cells Isolated from Menstrual Blood by Adherence

**DOI:** 10.1155/2016/3573846

**Published:** 2015-11-23

**Authors:** Xue Du, Qing Yuan, Ye Qu, Yuan Zhou, Jia Bei

**Affiliations:** ^1^Department of Obstetrics & Gynecology, General Hospital, Tianjin Medical University, Tianjin 300052, China; ^2^Department of Pathogenic Biology and Immunology, Logistics College of Chinese People's Armed Police Forces, Tianjin 300309, China; ^3^State Key Laboratory of Experimental Hematology, Institute of Hematology and Blood Diseases Hospital, Chinese Academy of Medical Sciences and Peking Union Medical College, Tianjin 300020, China; ^4^Department of Biomedical Engineering, University of Dundee, Dundee DD1 4HN, UK

## Abstract

*Objective*. To find a convenient and efficient way to isolate MSCs from human menstrual blood and to investigate their biological characteristics, proliferative capacity, and secretion levels. *Methods*. MSCs were isolated from menstrual blood of 3 healthy women using adherence. Cell immunological phenotype was examined by flow cytometry; the adipogenic, osteogenic, and chondrogenic differentiation of MSCs was examined by Oil-Red-O staining, ALP staining, and Alcian Blue staining, respectively; and the secretion of cytokines, including vascular endothelial growth factor (VEGF), hepatocyte growth factor (HGF), and insulin-like growth factor-1 (IGF-1), was detected using enzyme-linked immunosorbent assay. *Results*. MB-MSCs were successfully isolated from human menstrual blood using adherence. They were positive for CD73, CD105, CD29, and CD44, but negative for CD31 and CD45. The differentiated MB-MSCs were positive for ALP staining, Oil-Red-O staining, and Alcian Blue staining. In addition, they could secrete antiapoptotic cytokines, such as VEGF, IGF-1, and HGF. *Conclusion*. It is feasible to isolate MSCs from human menstrual blood, thus avoiding invasive procedures and ethical controversies. Adherence could be a promising alternative to the density gradient centrifugation for the isolation of MSCs from menstrual blood.

## 1. Introduction

Stem cells have been a topic of considerable interest in the biomedical field over recent years. Stem cells are undifferentiated cells that have a high proliferative potential, substantial self-renewal capacity, and the potential to differentiate into various specialized cell types. Therefore, stem cell therapy has emerged as a promising therapeutic strategy for traumas, immunodeficiency, and hereditary diseases due to their differentiation capacity and plasticity.

Stem cells can be broadly divided into embryonic and adult types. Embryonic stem cells (ESCs) are pluripotent stem cells derived from the inner cell mass of a blastocyst, an early-stage embryo. ESCs exhibit remarkable long-term proliferative potential and capacity for self-renewal, and they are also distinguished by their ability to differentiate into any cell type both* in vitro* and* in vivo*. Human ESCs were first derived from mouse embryos in 1981. However, major obstacles remain with the isolation and clinical use of ESCs due to teratoma formation and ethical controversies over the creation, usage, and destruction of human embryos.

Adult stem cells (ASCs) are undifferentiated cells found among differentiated cells of a specific tissue, including bone marrow, peripheral blood, umbilical cord blood, fat, brain, thymus, muscle, liver, and lung. It is well known that ASCs are typically in a dormant state under normal circumstances and start to proliferate and differentiate in response to pathological disorders and external stimuli. ASCs have a controlled behavior and are multipotent as compared to pluripotent capacity of ESCs, thus making them an attractive option for cell-based therapy. ASC treatments have been successfully used to treat Parkinson's disease [1], repair the kidney, and improve function in acute renal failure [2, 3] and liver injuries of different etiologies [4]. However, the use of ASCs can be limited by their availability, invasiveness of extraction, and in some cases limited proliferative capacity. Although mesenchymal stem cells (MSCs) from bone marrow have been regarded as good candidates for cell therapy, the sampling method is invasive through bone marrow aspiration. Despite their therapeutic potential, MSCs are very rare and a typical adult bone marrow aspirate yields very few MSCs (roughly 1 out of every 10,000 cells) [5].

Human menstrual blood might be an excellent source of ESCs. Chan et al. provided the first evidence for the existence of epithelial and stromal stem cells in human endometrium [6]. In 2004, Chan et al. [6] reported for the first time the isolation of stem cells directly from human endometrium. In 2007, Meng et al. [7] isolated MSCs from menstrual blood. Menstrual blood is easily accessible, renewable, and inexpensive. MSCs isolated from menstrual blood (MB-MSCs) may offer certain advantages, including noninvasive procedures,* in vitro* culture, and ethically nonproblematic availability. However, research on this new source of MB-MSCs is still in its infancy, and more research needs to be carried out on the isolation, secretion, and identification of MB-MSCs. In this study, MSCs were isolated from menstrual blood based on their strong adherence to plastic or glass, and their biological characteristics, proliferative capacity, and secretion levels were also investigated. The results of this study will help us to better understand the isolation of MSCs from human menstrual blood.

## 2. Materials and Methods

### 2.1. Collection of Menstrual Blood

Cell donation was approved by Local Ethical Committee for research purposes, and written informed consent was obtained from each donor. Infectious pathology was excluded by the HIV, HCV, and HBV tests. Menstrual blood was collected from three healthy female volunteers aged 25–35 years during the first 3 days of the cycle using a menstrual cup (Green Donna, Guangzhou Meifanle Rubber Products Co., Ltd., China). The menstrual cup was retained in the vagina for a mean of 3 hours (2–4 hours). The menstrual blood was transferred into phosphate buffered saline (PBS) containing 1% penicillin and streptomycin, maintained at 4°C, and transported to the laboratory within 24 h of collection.

### 2.2. Isolation and Culture of Human MB-MSCs

The menstrual blood was transferred to a 15 mL centrifuge, and red cells were depleted by Red Blood Cell Lysis Buffer (Sigma-Aldrich, St. Louis, MO, USA). Cells were counted by blood counting chamber. Cells were seeded at 5 × 10^5^ cells/well in 6-well plates (Corning, New York, USA) and cultured in DMEM-F12 supplemented with 10% FBS, streptomycin (100 U/mL) and penicillin (100 U/mL) (Gibco, Grand Island, NY) at 37°C in humidified air of 5% CO_2_. Culture medium was refreshed every 3-4 days. When the cells reached 80% confluence, they were passaged using 0.25% Trypsin (Gibco, Grand Island, NY, USA). All experiments were performed with cells at passage 3 from the 3 donors.

### 2.3. Immunophenotypic Analyses of MB-MSCS

Cells were harvested in 0.25% Trypsin and fixed in ice-cold 2% formaldehyde for 30 min. Then, the fixed cells were washed in PBS, adjusted to a density of 1 × 10^9^/L, and incubated with 1 *μ*L of antibodies (CD45 FITC, CD31 PE, CD44 APC-CY7, CD29 APC, CD105 Percp-Cy5.5, and CD73 PE) (BD Biosciences, San Jose, USA) at 4°C for 30 min. After that, cells were washed with PBS and centrifuged at 1500 r/min for 5 min. The supernatant was discarded, and the cells were resuspended with PBS. The antibody-labeled cells were analyzed using FACSCalibur flow cytometry (BD Biosciences, San Jose, CA), and the results were processed using Cell Quest.

### 2.4. Multilineage Differentiation of MB-MSCS

MB-MSCs were differentiated into adipogenic, chondrogenic, and osteogenic lineages to assess their differentiation capacities* in vitro*.

#### 2.4.1. Adipogenic Differentiation

MB-MSCs were seeded at 2 × 10^5^ cells/well in a 6-well chamber slide with 3 mL media per well. Upon reaching 90% confluence, the medium was replaced with adipogenic medium containing DMEM-F12, 10% FBS, 10^−6^ mol/L dexamethasone, 0.5 mmol/L isobutyl methylxanthine, 10 *μ*g/mL insulin, and 60 *μ*mol/L indomethacin (Sigma-Aldrich, St. Louis, MO, USA) and cultured for 14 days with medium changes every 3-4 days.

The adipogenic capacity of MB-MSCs was evaluated by Oil-Red-O staining. Cells were fixed with 4% paraformaldehyde for 30 min, washed with water three times, and then incubated for 30 min with a filtered 0.5% Oil-Red (Sigma-Aldrich Corp., St. Louis, MO, USA) solution in 60% isopropanol. Cells were washed with water several times prior to photographing. Oil-Red O-positive cells were determined by cytoplasmic fat globule cells.

#### 2.4.2. Osteogenic Differentiation

MB-MSCs were seeded at 4 × 10^5^ cells/well in a 6-well chamber slide with 3 mL media per well. After cells adhered overnight, the medium was replaced with osteogenic medium containing DMEM-F12, 10% FBS, 10^−8^ mol/L dexamethasone, 10 mmol/L *β*-glycerol phosphoric acid, and 100 mmol/L ascorbic acid (Sigma-Aldrich St. Louis, MO, USA), and cultures were continued for another 7 days.

The osteogenic capacity of MB-MSCs was evaluated by ALP staining. Cells were fixed in 4% paraformaldehyde for 30 s at room temperature, washed with water three times, and then incubated for 30 min with staining solution, which was prepared by dissolving Fast Blue RR Salt (Sigma-Aldrich, St. Louis, MO, USA) in 48 mL of ddH_2_O and 2 mL of Naphthol AS-MX Phosphate Alkaline (Sigma-Aldrich, St. Louis, MO, USA). Then, cells were washed with PBS and incubated for 30 min with Mayer's Hematoxylin (Sigma-Aldrich, St. Louis, MO, USA). The stained cells were washed and imaged under microscope. Purple staining indicated the synthesis of ALP by osteoblasts.

#### 2.4.3. Chondrogenic Differentiation

MB-MSCs were seeded at 4 × 10^5^ cells/mL in a 6-well chamber slide with 3 mL media per well. After cells adhered overnight, the medium was replaced with achondrogenic medium containing DMEM-F12, 10% FBS, 10^−8^ mol/L dexamethasone, 10 mmol/L *β*-glycerol phosphoric acid, 100 mmol/L ascorbic acid, and 10 ng/mL TGF *β*-3 (Sigma-Aldrich, St. Louis, MO, USA). Cultures were allowed to develop for 21 days with medium changes every 3-4 days and were stained by Alcian Blue. Briefly, cells were washed with PBS and fixed with 4% paraformaldehyde for 30 min. After fixation, cells were washed with PBS and stained with 1% Alcian Blue solution (Sigma-Aldrich Corp., St. Louis, MO) in the dark overnight at room temperature. Then, cells were washed three times with PBS and visualized under a light microscope. Blue staining indicated synthesis of proteoglycans by chondrocytes.

### 2.5. Cell Growth Curve Analysis

Cells at P3, P10, and P15 were seeded at 1.0 × 10^3^ cells/well in 96-well plates (Corning, New York, USA) and cultured in DMEM-F12 supplemented with 10% FBS, 1% streptomycin, and 1% penicillin. After 1, 3, 5, 7, and 9 days, cell proliferation was analyzed by a CCK-8 kit. Briefly, 10% CCK-8 solution (Beyotime, Shanghai, China) was added to each culture and incubated for 4 h. Then, optical densities (OD) were measured at 450 nm using an ELISA plate reader (BioTek, Highland Park, USA).

### 2.6. Enzyme-Linked Immunosorbent Assay (ELISA)

MB-MSCs were seeded at 5.0 × 10^5^ cells/well in 6-well plates. Complete medium replacement was performed when cells reached 90% confluence. Within 48 h, the cells were spun down in a centrifuge, and the supernatant was collected to measure the secretion of growth factors, including vascular endothelial growth factor (VEGF), hepatocyte growth factor (HGF), and insulin-like growth factor-1 (IGF-1) according to manufacturer's protocols (Ruikang Bo, Tianjin, China). Optical densities (OD) were measured at 450 nm using an ELISA plate reader.

### 2.7. Statistical Analysis

All analyses were performed using SPSS statistical software (version 13.0; SPSS Inc., Chicago, IL). Results are expressed as mean ± standard deviation. Significance of difference between groups was assessed by *t*-test or ANOVA, and regression analysis was performed. A one-tail *P* value of <0.05 was considered as statistically significant.

## 3. Results

### 3.1. Isolation and Culture of MB-MSCs

**Figure 1:**
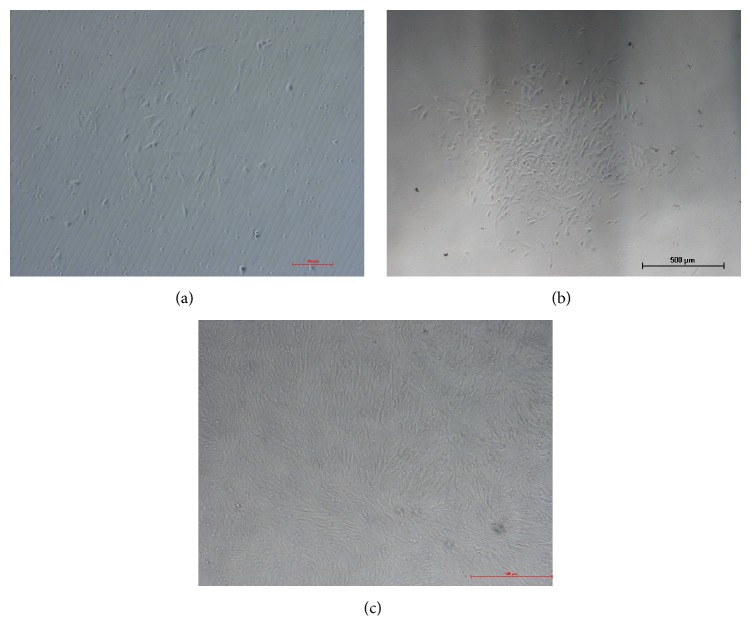
The morphology of cultured MB-MSCs. (a) The morphology of cultured MB-MSCs at 6 day. (b) Primary cell culture showing initial colony forming unit-fibroblast (CFU-F) on day 8. (c) Cells reached 85–90% confluence at passage 3.

MB-MSCs adhered on day 6 after two changes of medium, and some nonadherent hematopoietic cells were present in the culture (Figure 1(a)). After initial plating was removed with sequential exchange of culture medium, many colony forming unit-fibroblast (CFU-F) colonies were formed after 8 d of incubation (Figure 1(b)). Cell morphology varied from spindle-shaped to triangular arranged in an unorganized pattern. The number of colonies increased gradually to 80% confluence after 15 d of seeding. However, after 2-3 subcultures, the cells reached 90% confluence only for 5 d (Figure 1(c)).

### 3.2. Cell Surface Antigen Expression

**Figure 2:**
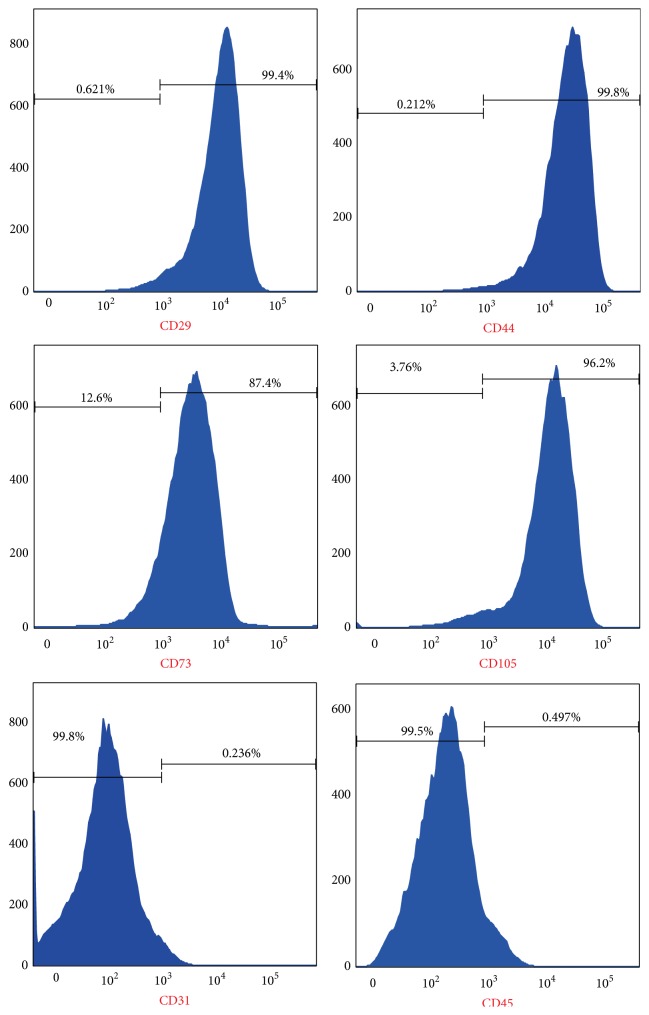
Expression of surface markers of P3 MB-MSCs. Percentages are referring to the average positivity for each marker.

The immunophenotypic analysis (Figure 2) showed that P3 MB-MSCs were positive for CD73 (87.4%), CD105 (96.2%), CD29 (99.4%), and CD44 (99.8%), but negative for CD45 (0.497%) and CD31 (0.236%).

### 3.3. Multilineage Differentiation of MB-MSCSs

**Figure 3:**
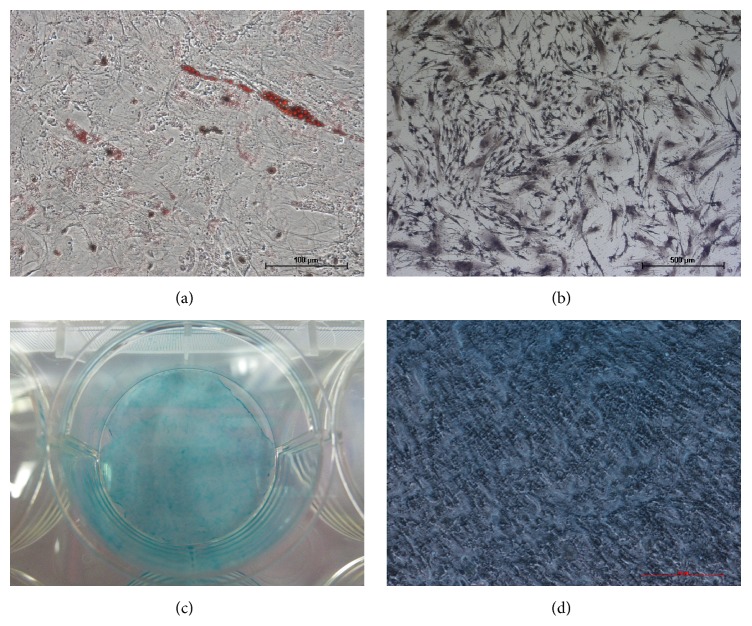
Multipotential differentiation of MB-MSCs. (a) Adipocytic differentiation. Red indicated lipid vacuoles stained by Oil-Red. (b) Osteocytic differentiation. Purple indicated alkaline phosphatases stained by ALP staining. (c, d) Chondrocytic differentiation. Blue indicated extracellular matrix component glycosaminoglycans stained by Alcian Blue.

MB-MSCs cultured in medium supplemented with differentiation factors could differentiate into mesenchymal lineages, such as osteoblasts, adipocytes, and chondrocytes. The adipogenic differentiation was demonstrated by Oil-Red-O staining on day 14 (Figure 3(a)), and adipocytes were easily identified morphologically by the presence of lipid vacuoles in the cytoplasm. During osteogenic stimulation, MB-MSCs continued to proliferate and changed in morphology. On day 14, almost all alkaline phosphatases in the cytoplasm were stained to be purple (Figure 3(b)). The chondrogenic potential of MB-MSCs was characterized by Alcian Blue staining after 21 days of culture, which revealed a deposition of proteoglycans (Figures 3(c) and 3(d)).

### 3.4. Cell Proliferation Assay

**Figure 4:**
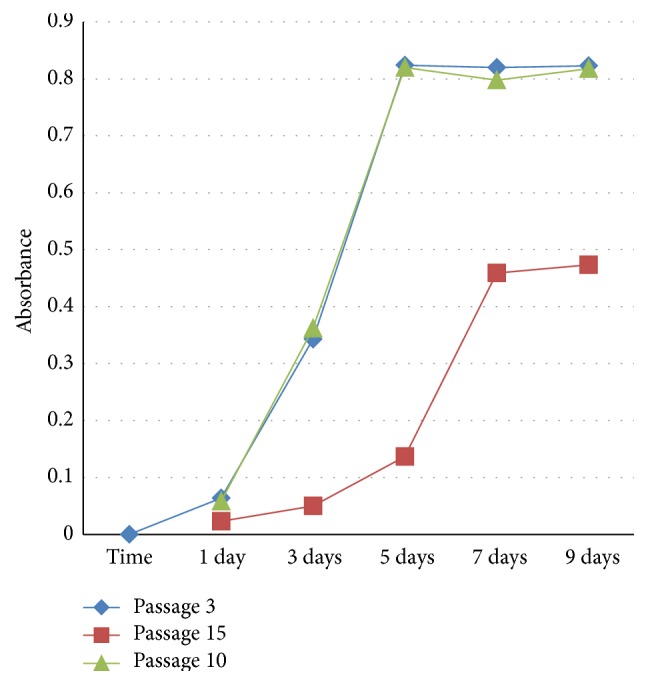
Growth curves of subcultured MB-MSCs.

The growth of MB-MSCs was analyzed at early (P3 and P10) and late (P15) passages, and cell proliferation was monitored for 9 days after seeding (Figure 4). The growth of P3, P10, and P15 MB-MSCs all showed a logistic (S-shaped) growth pattern that increased very slowly during the first 1-2 days, followed by an exponential rise from day 2 till day 6 and a stationary phase thereafter. No significant difference in absorbance was observed between P3 and P10 MB-MSCs, but the absorbance of P15 passage was significantly lower. Thus, it could be concluded that there was no difference in cell proliferation between P3 and P10 MB-MSCs, indicating that MB-MSCs still had a strong proliferative potential at P10.

### 3.5. Secretion of Growth Factors

The ELISA analysis showed that the secretion of growth factors was undetectable in the control group (data not shown), but a large amount of angiogenic and antiapoptotic factors was secreted from MB-MSCs after 3 days of culture, which were 122.953 ± 4.377 pg/mL for VEGF, 4926.938 ± 890.475 pg/mL for HGF, and 1.77 ± 0.027 ng/mL for IGF-1, respectively.

## 4. Discussion

Human endometrium is composed of functional and basal layer. The endometrium is about 0.5–1 mm following menstruation, whereas it is about 10–12 mm at the end of the cycle. Thus, it is hypothesized that endometrial stem/progenitor cells may play a key role in mediating endometrial repair and subsequent tissue regeneration following menstruation [8]. Chan et al. provided the first evidence for the existence of ESCs in endometrium [6]. However, a potential problem with acquisition of human endometrium is that it is a very invasive procedure. In the recent years, a number of studies have shown that menstrual blood contains a unique population of cells with properties similar to ASCs [7, 9, 10]. An advantage of MB-MSCs is their easy availability, thus avoiding invasive procedures and ethical controversies. In addition, MB-MSCs hold great promise to avoid teratoma formation and immune response of ESCs, and they are also highly expandable* in vitro* and possess pluripotency. For these reasons, MB-MSCs are increasingly used in a variety of clinical applications, such as stroke [11] and myocardial infarction [12].

MSCs can be isolated from different sources. MSCs may be derived from residual fetal stem cells [13], and there are several lines of evidence indicating that bone marrow-derived stem cells may also populate the endometrium [14, 15]. It is postulated that most MSCs are located in blood before birth, and some are found in perivascular locations with a unique potential to differentiate into mesenchymal tissue. Later MSCs can migrate to liver, spleen, and bone marrow. After birth, MSCs mainly reside in bone marrow but may also be present in menstrual blood, cord blood, fat, brain, thymus, muscle, liver, and lung [16–19]. Bone marrow stem cells home in on sites of tissue damage and incorporate into various organs, contributing to angiogenesis and/or transdifferentiating into the cells of the new tissue in which they reside [20].

In this study, we have shown that MSCs could be isolated from menstrual blood based on their strong adherence properties. Nonadherent endothelial cells and fibroblasts were removed with sequential change of culture medium. By day 6, cells adhered, and cell morphology varied from spindle-shaped to triangular. The spindle-shaped cells accounted for the majority of cells and had CFU activity by forming large and small CFU-F. It was also observed that cells expressed high levels of CD73, CD90, CD29, CD73, and CD90, the main stem cell markers [21]. But they lacked CD45, a hematopoietic stem cell marker, and CD31, a vascular endothelial cell marker, which was also in agreement with previous studies [7, 9].

As no ideal marker of MB-MSCs has been discovered, there is a need for further identification of multilineage differentiation. MSCs cultured* in vitro* in adipogenic, osteogenic, and chondrogenic medium differentiate into adipocytes, osteocytes, and chondrocytes, respectively. Adipogenic differentiation was evidenced by the presence of lipid vacuoles in the cytoplasm by Oil-Red-O staining. Osteogenic differentiation was evidenced by ALP staining. Differentiation of chondrocytes from MB-MSCs was examined cytochemically with respect to the presence of proteoglycan (Alcian Blue positive). In this study, we found that not all MB-MSCs could differentiate, probably due to the stimulation of chemical reagents, or that MSCs were in different stages of differentiation. It has been demonstrated that most MB-MSCs had the ability to differentiate into cardiac muscle cells and myocytes [22, 23]. Finally, we showed that MB-MSCs had multilineage differentiation potential.

In this study, MSCs were isolated from menstrual blood using adherence of whole menstrual blood, which is capable of maintaining the stemness and differentiation ability of MSCs. In addition, it is more convenient and efficient than Ficoll density gradient centrifugation. Density gradient centrifugation is a common method used for the separation of MB-MSCs based on their different densities [10]. However, there are a variety of cells in menstrual blood with different densities; thus, the use of density gradient centrifugation can result in loss of MB-MSCs. As such, we used adherence for isolation of MSCs from menstrual blood. It allows for quick adherence and cell growth, and nonadherent hematopoietic cells and dead cells can be removed with the change of culture medium. In addition, there is no significant difference in purity. It has been suggested that a culture system using a small amount of fibroblasts as feeder cells allows the production and survival of embryonic stem cells due to the synergistic effect of the secretion of growth factors [24]. Thus, adherence could be a promising alternative to the density gradient centrifugation for the isolation of MSCs from menstrual blood.

We also investigated the growth of MB-MSCs to characterize their proliferative capacity. It was observed that MB-MSCs could be cultured for 10–15 sequential passages (more than 20 months). After freezing and thawing, they still preserved proliferation capacity and clonogenicity. The results of this study showed that MB-MSCs could be subcultured for up to 35 passages, which was in agreement with the findings of Allickson et al. [25], indicating that MB-MSCs were highly proliferative. This may be associated with telomerase activity [26]. Telomerases serve to protect chromosome ends from end-to-end fusion, recombination, and degradation. It has been shown that knock-out of telomerase leads to telomere shortening and chromosomal abnormalities, thus reducing the proliferative capacity of stem cells [27].

We found that MB-MSCs could secrete VEGF, HGF, and IGF-1 that played a key role in tissue repair and regeneration. Rehman et al. [28] showed that adipose tissue-derived MSCs could secrete antiapoptotic factors, such as VEGF, HGF, and IGF-1. Tögel et al. [29] confirmed that these factors improved kidney function through the bypass secretion, rather than differentiating into target cells. Fu et al. [30] showed that MSCs produced a wide array of cytokines, such as VEGF, IGF-1, and HGF, and direct injection of BM-MSCs into ovary enhanced ovarian function in rats with chemotherapy-induced ovarian damage. These effects appear to be mediated by the paracrine mechanisms of cytokines. MSCs are present in bone marrow, peripheral blood, cord blood, fat, brain, thymus, muscle, liver, and lung, but the use of MSCs could be limited due to invasiveness of extraction and proliferative capacity. It is clear that MSCs from menstrual blood offer certain advantages including noninvasive and ethically acceptable availability; thus, they may be a practical solution for stem cell therapy.

In conclusion, we successfully isolated MSCs from menstrual blood using adherence and investigated their biological characteristics, proliferative capacity and secretion levels. MB-MSCs showed a high multilineage potential and clonogenic activity and secreted a large amount of VEGF, HGF, and IGF-1. These factors might play a key role in tissue repair and regeneration. In comparison with MSCs from other sources, a significant advantage of MB-MSCs is their easy availability, thus avoiding invasive procedures and ethical controversies. Isolation of MSCs from menstrual blood using adherence is very simple and time saving. However, there are still many problems to be solved, such as surface markers with sufficient specificity to identify MB-MSCs and the source of MB-MSCs. We believe that this cell population could become a practical solution for autologous stem cell therapy.
